# Synthesis, characterization and antimicrobial activity applications of grafted copolymer alginate-*g*-poly(*N*-vinyl imidazole)†

**DOI:** 10.1039/d1ra01874d

**Published:** 2021-03-19

**Authors:** Soliman Mehawed Abdellatif Soliman, Mohamed Fathi Sanad, Ahmed Esmail Shalan

**Affiliations:** Chemistry Department, Faculty of Science, Cairo University 12613 Giza Egypt sabdellatif@sci.cu.edu.eg; Central Metallurgical Research and Development Institute (CMRDI) P.O. Box 87 Helwan Cairo 11421 Egypt; FabLab, Centre for Emerging Learning Technologies (CELT), Electrical Engineering Department, The British University in Egypt (BUE) Cairo 11387 Egypt; BCMaterials, Basque Center for Materials, Applications and Nanostructures Martina Casiano, UPV/EHU Science Park, Barrio Sarriena s/n Leioa 48940 Spain ahmed.shalan@bcmaterials.net a.shalan133@gmail.com

## Abstract

*N*-Vinyl imidazole was grafted onto sodium alginate (P*N*VI-*g*-NaAlg) through a free radical polymerization technique in aqueous solution using potassium persulfate (K_2_S_2_O_8_, KPS) initiator material. The conditions of the grafting process onto sodium alginate were adjusted to obtain a grafted copolymer with a high percentage of poly(*N*-vinyl imidazole). The prepared grafted copolymer sodium alginate (NaAlg-*g*-P*N*VI), with high percentage yield, was investigated and characterized under certain conditions in order to detect its antibacterial effect. The prepared grafted copolymer was considered by means of several systems such as Fourier-Transform Infrared spectroscopy (FT-IR), ^1^H NMR spectroscopy and thermal analysis. The change in the morphology of the alginate distinguished after the grafting process was confirmed using a Scanning Electron Microscope (SEM). The biological activity of the grafted material was considered using *Escherichia coli*, *Neisseria gonorrhoeae* (Gram-negative), *Bacillus subtilis* (Gram-positive) and *Candida albicans* antifungal activities through the agar diffusion method. The obtained results show excellent improvement in antimicrobial activity of the alginate by grafting against *Bacillus subtilis*, *Escherichia coli*, *Neisseria gonorrhoeae*, and *Candida albicans*.

## Abstract 2

*N*-Vinyl imidazole was grafted onto sodium alginate (P*N*VI-*g*-NaAlg) through a free radical polymerization technique in aqueous solution using potassium persulfate (K_2_S_2_O_8_, KPS) initiator material.

## Graphical abstract

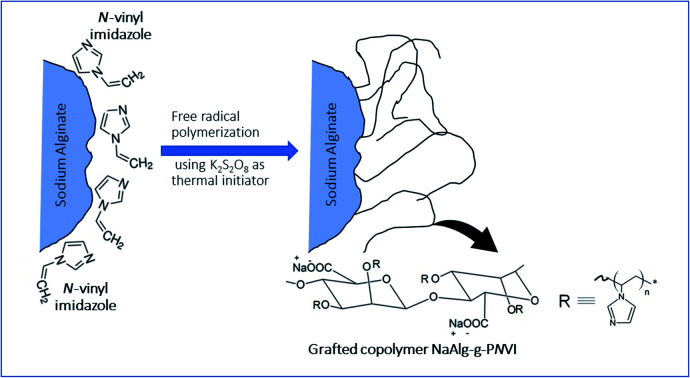

## 1. Introduction

Alginate is considered a anionic natural polysaccharide extracted from brown seaweed.^1^ It is also considered as a linear unbranched polymer that consists of different linked hexuronic acids which have been called β-(1,4)-d-mannuronopyranosyl (M) residues and α-(1,4)-l-guluronopyranosyl (G) residues. It has plenty of free hydroxyl and carboxyl groups dispersed along the backbone chain of the natural polymer. This dispersion of both characteristic groups enables alginate to be modified for further copolymerization reactions.^2^ Because of alginate's low toxicity, biocompatibility, and its low cost, further biomedical application has been recommended.^3^ The outstanding hemostatic properties of alginate are one of the best for hemostatic wound dressing applications. Alginate/poly(*g*-glutamic acid) composite microparticles have been synthesized from alginate using an emulsification–internal gelation method where a double structure network was noticed in the micro particles due to the ion chelation between Ca^2+^ and the carboxylic groups of alginate.^4^ Chemical modification of the hydroxyl and carboxyl groups along the backbone chain of alginate such as oxidation, sulfation and esterification reactions or either amidation reaction were possible.^5^ Furthermore, alginate has a wide remarkable role in food packaging due to its low toxicity, which is observed in the fabrication of poly(methylmethacrylate-*g*-sodium alginate) composite films. In addition, it was observed that the improvement of the thermal stability and water resistance of those films can be utilized for better food packaging purposes.^6^ Grafted copolymer alginate-*g*-poly(vinyl caprolactam) has been fabricated for 5-fluorouracil controlled delivery.^7^ Among various types of polymers, *N*-vinyl imidazole has been highly recommended for different polymerization and copolymerization reactions due to its polymerization activity and high thermodynamic activity, which increase the possibility to fabricate different structures and functionalities of various polymers. These prominent properties of *N*-vinyl imidazole can effect ionic strength and change in pH and swelling ability of the medium.^8^ In addition, *N*-vinyl imidazole is working as a significant functional monomer by reason of its noticeable biocompatibility, metal chelation, outstanding antimicrobial activity, and protein adsorption ability.^9^ Moreover, poly(*N*-vinyl imidazole) is non-toxic polymer for use in a wide range of biomedical applications such as gene delivery,^10^ and drug delivery system.^11^ Copolymers of *N*-vinyl imidazole have great anxiety in macromolecular science and this is based on the ability to synthetic new biopolymers that have gigantic biological activity.^11–19^ Moreover, *N*-vinyl imidazole was also important in the fabrication of biologically active compounds due to its possessive properties in the physiological pH range.^20^*N*-Vinyl imidazole was grafted onto different natural polymer to improve the antimicrobial activity such as chitosan,^21^ carboxymethyl chitosan,^22^ carboxymethyl starch of antibacterial activity against *Xanthomonas perforans* and *Xanthomonas oryzae* and carboxymethyl cellulose to be used as drug delivery system.^23,24^

In the current work, we study the effect of different concentrations of *N*-vinyl imidazole and potassium persulfate (K_2_S_2_O_8_, KPS) onto the grafting process onto sodium alginate. *N*-Vinyl imidazole grafted onto sodium alginate was prepared, characterized *via* different techniques and the antibacterial activity effect of the synthesized materials was tested against different species of bacteria and fungus.

## 2. Experimental section

### 2.1 Materials

Sodium alginate was acquired *via* (Nice Chemicals Pvt. Ltd.) with viscosity of 1000–1200 cPs. *N*-Vinyl imidazole (*N*VI) was obtained from Merck (Schuchardt OHG, Hohenbrunn, Germany) company. Potassium persulfate (KPS) was purchased from (Sigma-Aldrich, USA). No further purification was needed for the other reagents and solvents that were used in the current study as they were bought with the standard analytical grades.

### 2.2 Characterization techniques

Grafted copolymer alginate-*g*-P*N*VI was proved by ^1^H NMR spectroscopy (Varian Mercury VX-300) in D_2_O solvent. FT-IR spectra of sodium alginate and its grafted copolymer were detected, within the wave number range of 4000–600 cm^−1^ at 25 °C, *via* TENSOR Testcan Shimadzu IR-spectrophotometer (model 8000) using KBr pellets. Furthermore, the thermal properties were investigated through Shimadzu Thermogravimetric Analyzer (TGA-50H) where the prepared samples were heated from 20–500 °C under N_2_ atmosphere (with flow rate of 25 mL min^−1^ and heating rate of 10 °C min^−1^). In addition, the morphology of tested samples was shown using Quanta 250 FEG microanalyzer at 30 kV scanning electron microscope (SEM, JOEL S150A) (with field emission gun). Samples were handled by covering of dry samples on the substrate with gold (Au) layer with thickness of almost 100 nm using ion sputter coating unit for 5 min.

### 2.3 Antibacterial assays and antimicrobial activity for P*N*VI-*g*-NaAlg copolymers

Antimicrobial activity of the tested samples was determined using a modified Kirby–Bauer disc diffusion method.^25^ Briefly, 100 μL of the test bacteria/fungi were grown in 10 mL of fresh media until they reached a count of approximately 10^7^ cells per mL for bacteria or 10^5^ cells per mL for fungi.^26^ Furthermore, 100 μL of microbial suspension was spread onto agar plates corresponding to the broth in which they were maintained. All the sterilization and disinfection conditions were followed up for the antimicrobial activity actions. All the samples were sterilized using ethyl acetate and acetone before antibacterial tests. Furthermore, the antimicrobial activity of the samples was tested and implemented by a modified Kirby–Bauer disc diffusion process as studied elsewhere.^22^ Plates vaccinated with filamentous fungi as *Aspergillus flavus* at 25 °C for 48 hours; Gram (+) bacteria (*Bacillus subtilis*, ATCC: 6051) Gram (−) bacteria (*Escherichia coli*, ATCC: 11775 and *Neisseria gonorrhoeae*, ATCC: 19424), as bacterial cell suspension that diluted to an arranged cell concentration *via* using sterilized distilled saline solution in order to be utilized for the antibacterial assessments for the grafted copolymers. Moreover, the turbidity comparison method was utilized to detect the viable cell number in the cell suspension. The polymer/bacterial suspension arrangements were incubated at 35–37 °C for 24–48 hours and yeast as *Candida albicans* raised at 30 °C for 24–48 hours and, at that point, the diameters of the inhibition zones were detected in millimeters depending on the following eqn (1):^27^1
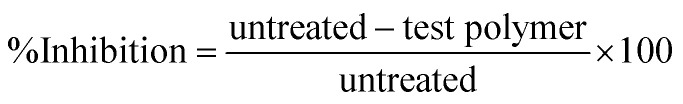


### 2.4 Grafting of *N*-vinyl imidazole (*N*VI) onto sodium alginate

**Fig. 1:**
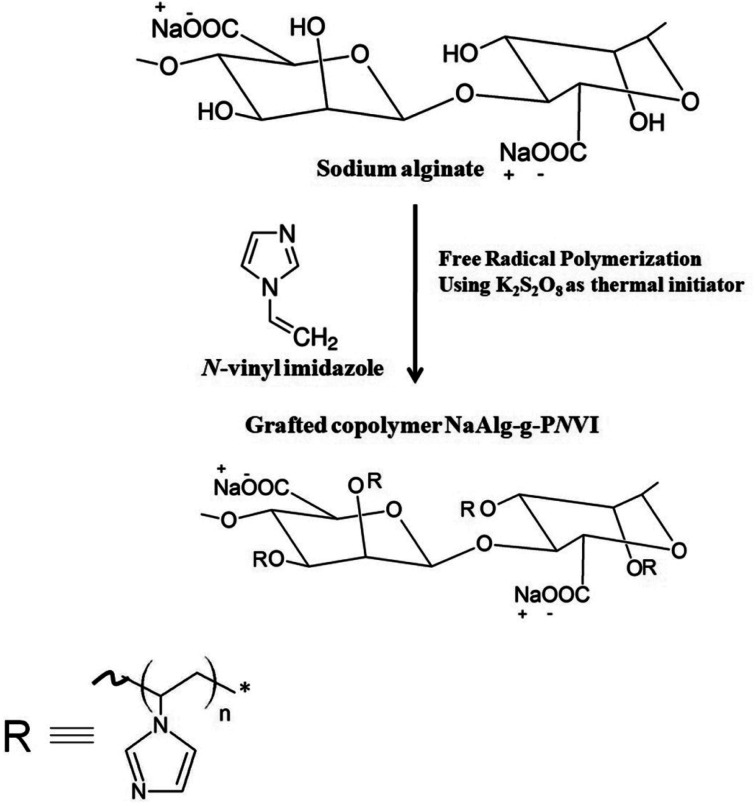
The grafting process of *N*-vinyl imidazole onto sodium alginate through free radical polymerization.

0.5 gm (*W*_NaAlg_) of sodium alginate was added to 20 mL of distilled water with vigorous stirring under nitrogen gas flow in three-necked bottom flask. Then, defiant weights (*W*_*N*VI_) of *N*-vinyl imidazole (*N*VI) was added to the solution and followed by adding 4 × 10^−2^ mol L^−1^ from the solution of potassium persulfate (KPS) under the nitrogen atmosphere were added and heated at temperature of 60 °C for 4 h (Fig. 1). The homogeneous grafted alginate copolymer mixture is precipitated in cold acetone then dried and weighted (*W*_total_). Furthermore, ethanol was used to extract the non-grafted formed homopolymer (poly(*N*-vinyl imidazole)) from the crude grafted copolymer *via* soxhlet device extraction process for 8 h. In addition, the grafted copolymer “P*N*VI-*g*-NaAlg” was dried in an air oven at 40 °C until constant weight (*W*_NaAlg-*g*-P*N*VI_) and the grafting process parameters were detected based on the next eqn (2)–(4).2
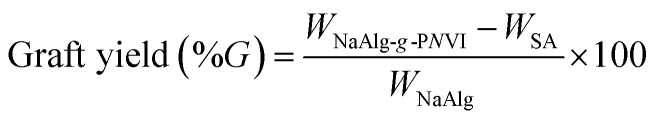

3 %*H* = ((*W*_total_ − *W*_NaAlg-*g*-P*N*VI_)/*W*_*N*VI_) × 100

4
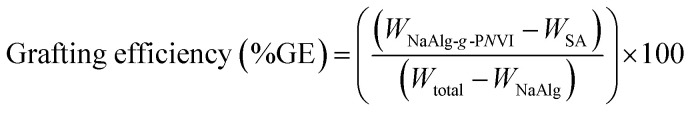


## 3. Result and discussion

In this section, studying the effect of concentrations of *N*-vinyl imidazole and K_2_S_2_O_8_ onto grafting of sodium alginate.

### 3.1 K_2_S_2_O_8_ concentration effect

**Fig. 2:**
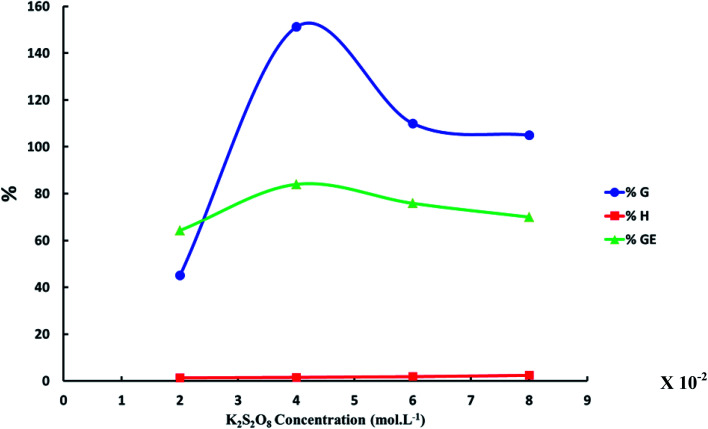
The relation between the increase of K_2_S_2_O_8_ concentration and graft yield (*G*%), graft efficiency (GE%) and homopolymer percent (*H*%). ([*N*-Vinyl imidazole] = 1.5 mol L^−1^, reaction time = 4 h and reaction temperature = 60 °C).

Effect of initiator concentration (K_2_S_2_O_8_) on the grafting of *N*-vinyl imidazole from sodium alginate was estimated with keeping temperature at 60 °C, which is temperature decomposition of potassium persulfate for 4 hours and concentration of *N*-vinyl imidazole equal 1.5 mol L^−1^. We observed the optimum concentration of KPS to be equal to 4 × 10^−2^ mol L^−1^ that gives highest graft yield (*G*%) and graft efficiency (GE%) as founded in Fig. 2. The highest concentrations of KPS give lower graft yield (*G*%) due to both initiation and termination reactions occurring to initiators.

### 3.2 *N*-Vinyl imidazole concentration effect

**Fig. 3:**
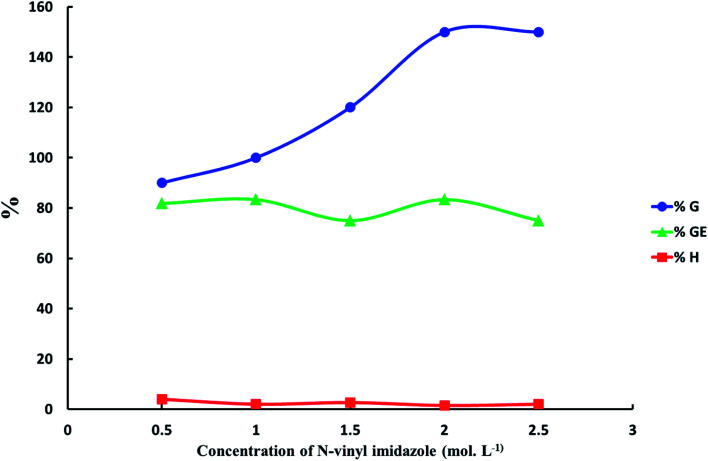
The relation between the increasing of *N*-vinyl imidazole concentration and graft yield (*G*%), graft efficiency (GE%) and homopolymer percent (*H*%). ([K_2_S_2_O_8_] = 4 × 10^−2^ mol L^−1^, reaction time = 4 h and reaction temperature = 60 °C).

*N*-Vinyl imidazole concentration effect on grafting of *N*-vinyl imidazole from sodium alginate was estimated with keeping temperature at 60 °C, which is temperature decomposition of potassium persulfate for 4 hours, and using optimum concentration of initiator equal 4 × 10^−2^ mol L^−1^. The maximum graft yield (*G*%) was achieved at *N*-vinyl imidazole concentration equal 2 mol L^−1^ as shown in Fig. 3.

### 3.3 Characterization of the synthesized grafted copolymers

#### 3.3.1 ^1^H NMR

As shown in the ^1^H-NMR spectrum of grafted copolymer alginate-*g*-P*N*VI, characteristic peaks of alginate appeared about 3.5–4.5 ppm and anomeric proton interferes with peak of D_2_O (Fig. S1, in the ESI†). In addition to characteristic peaks of poly(*N*-vinyl imidazole) as protons of backbone CH_2_ and CH appear at 1.5 and 2–2.9 ppm, respectively. Peaks of imidazole ring observed over range 6.9 to 7.4 ppm.^28^

#### 3.3.2 FT-IR measurements

**Fig. 4:**
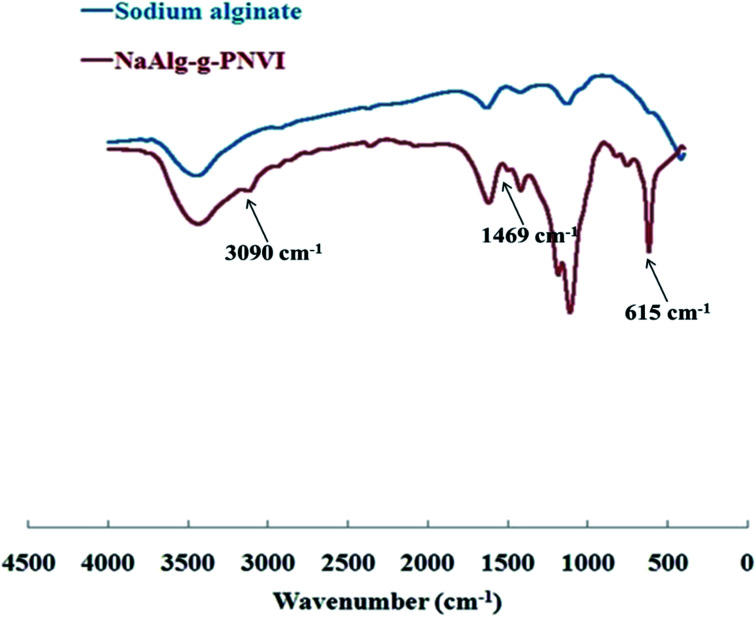
FT-IR of sodium alginate and its grafted copolymer NaAlg-*g*-P*N*VI.

The FT-IR spectrum of sodium alginate, which showed numerous absorption bands at 1100, 1017 and 800 cm^−1^, is characteristic of the polysaccharide structure as illustrated in Fig. 4. The broad absorption band at 3500–3200 cm^−1^, is on account of the stretching frequency of the –OH group, while at 1030 cm^−1^ is owing to the bending vibrations of –OH group, and at 2925 cm^−1^ is due to the incidence of the –CH stretching vibration. Furthermore, two additional peaks at 1420 cm^−1^ and 1620 cm^−1^ that show a symmetric and asymmetric stretching vibration of COO^−^ groups have appeared and been detected. Furthermore, the IR spectrum of the grafted copolymer PVI-*g*-NaAlg (Fig. 4) also shows an overlapped broadband at 3400–3600 cm^−1^ that is because of the existence of –OH stretching. For the NaAlg-*g*-P*N*VI, the absorption bands at 3100 and 3090 cm^−1^ were imputed to the stretching vibration of the –CH in imidazole ring and CH_2_ in the backbone, respectively. At 1400 cm^−1^, the peak appears due to the presence of the bending vibration of the CH_2_ group. The absorption band at 1469 cm^−1^ was assigned to the stretching vibration of the C=C and C=N group in the imidazole ring. In addition, the strong peak at 615 cm^−1^ is attributable to the deformation out-of-plane-bending of imidazole ring.^28^ The distinctive peaks of vinyl imidazole revealed in the FT-IR spectrum of graft copolymer spectrum NaAlg-*g*-P*N*VI proved that successful grafting of vinyl imidazole onto sodium alginate.

### 3.4 Thermogravimetric analysis of the prepared grafted copolymers

**Fig. 5:**
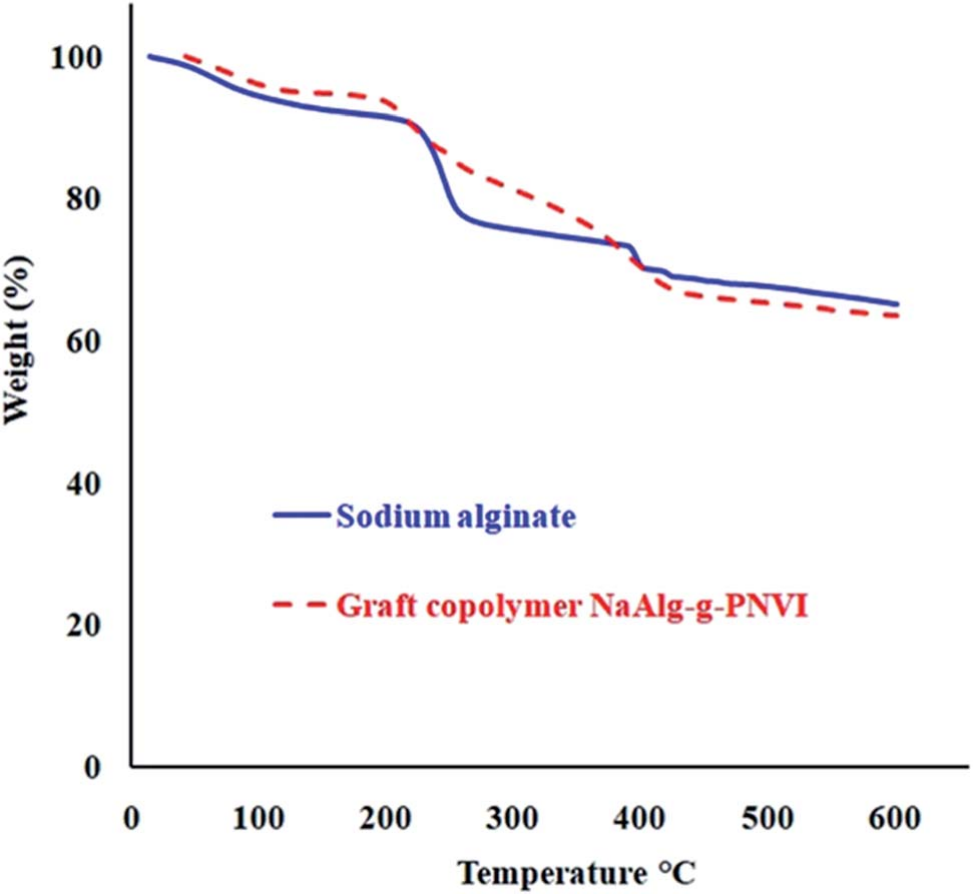
Thermogravimetric analyses of sodium alginate and its grafted copolymer NaAlg-*g*-P*N*VI.

Thermogravimetric analysis (TGA) of sodium alginate indicates the existence of two corruption forms. One procedure happened over the zone of temperature from 225–251 °C that coming out because of the decarboxylation and release of CO_2_, while the second debasement is seen to start from 390 °C because of the depolymerization of the polymer which prompts carbonaceous buildup and Na_2_CO_3_.^29–31^ Furthermore, the grafted copolymer NaAlg-*g*-P*N*VI was subjected to thermogravimetric analysis in order to investigate its thermal stability compared with sodium alginate itself. According to the information given in Fig. 5, there were two main weight loss steps in the TGA curve. Two steps occur at an approximately same temperature range in case of sodium alginate TGA curve. The initial decomposition temperature of sodium alginate and its grafted copolymer are 225 and 200 °C, respectively. Subsequently, the grafting of P*N*VI onto sodium alginate doesn't affect the initial degradation temperature of sodium alginate. On another hand, the TGA curve of grafted copolymer shows the rate of degradation is slow compared to the rate of degradation of sodium alginate (Table 1). It was noticed that almost 20% weight loss occurs at 251 and 316 °C in the case of sodium alginate and its copolymer NaAlg-*g*-P*N*VI, respectively.

**Table 1:** Thermal behavior of sodium alginate and its grafted copolymer NaAlg-*g*-P*N*VI

Polymer/hydrogel	Initial decomposition temp. (IDT) (°C)	Temp. wt. loss 20%	Wt. loss (%) at 350 °C
Sodium alginate	225	253	26
NaAlg-*g*-P*N*VI	200	316	23

### 3.5 Microstructure detection of the prepared grafted copolymers

**Fig. 6:**
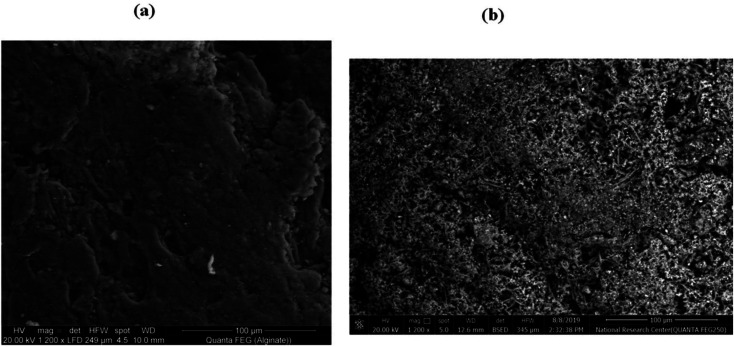
SEM pictures of (a) sodium alginate and (b) its grafted copolymer NaAlg-*g*-P*N*VI.

Scanning electron microscope (SEM) of sodium alginate and grafted copolymer NaAlg-*g*-P*N*VI (%*G* = 120%) are presented in Fig. 6a and b. The surface of native sodium alginate was found to be smooth that completely changed as compared with the surface of the grafted sample as found in Fig. 6a. In the obtained image of the grafted copolymer, it was found that a poly(*N*-vinyl imidazole) grafted chain has covered all the surface of sodium alginate (Fig. 6b). The robes structure of graft polymer P*N*VI onto sodium alginate surface leads to porous surface. This new surface structure expected to improve the antimicrobial activity of the copolymer alginate-*g*-P*N*VI.^32^ Additionally, SEM with higher magnification for the grafted copolymer NaAlg-*g*-P*N*VI were checked to confirm the formed structure with mesoporous surface (Fig. S2a, ESI†). Besides, the average particle size of the obtained materials was calculated using ImageJ software and founded to be in the range of 300–600 nm (Fig. S2b, ESI†).

### 3.6 Antimicrobial activity of sodium alginate and its NaAlg-*g*-P*N*VI copolymers

The antimicrobial activity of sodium alginate and its copolymers is based on different parameters like the pH of the medium, the temperature of incubation, *etc.* Furthermore, the antimicrobial activity of sodium alginate and its copolymers was assumed *via* numerous hypotheses, such as the change in the cell permeability because of the connections between the sodium alginate and the electronegative charges on the cell surfaces. Furthermore, the gained contact directs to the drip of intracellular electrolytes and proteinaceous constituents.^33–37^ On the other hand, additional directions were detected through the contact of diffused hydrolysis species with microbial DNA that may cause the embarrassment of mRNA in addition to the existence of protein.^38–40^

**Fig. 7:**
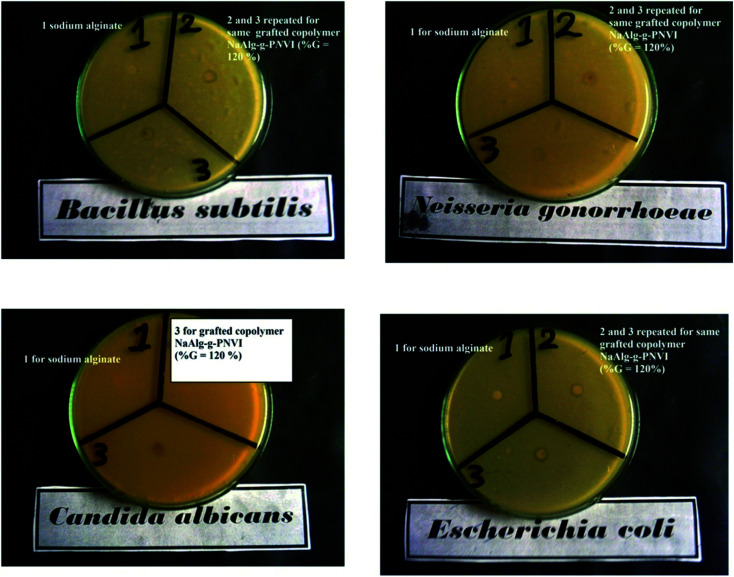
Antimicrobial activity of the sodium alginate (1) and its grafted copolymer (repeated 2 and 3) against Gram (−) bacteria (*Escherichia coli* and *Neisseria gonorrhoeae*), Gram (+) bacteria (*Bacillus subtilis*) and fungi (*Candida albicans*).

Data from Table 2 revealed the exposure effect of Gram-negative bacteria (*Escherichia coli* and *Neisseria gonorrhoeae*) and Gram-positive bacteria (*Bacillus subtilis*) towards sodium alginate and grafted NaAlg-*g*-P*N*VI copolymer. Concerning Gram-positive bacteria, alginate has no activity on a *Bacillus subtilis*, while grafted copolymer NaAlg-*g*-P*N*VI with %*G* = 120% showed remarkable inhibition zone diameter of 10 mm mg^−1^ as illustrated in Fig. 7. The different inhibition zones diameters observed in case of alginate and its grafted copolymer NaAlg-*g*-P*N*VI (%*G* = 120%) were illustrated in Table 2 in detail. The remarkable inhibition zone diameter of the grafted copolymer NaAlg-*g*-P*N*VI (*G*% = 120%) could be elucidated through the incidence of imidazole rings that have auspicious antibacterial features that showing minor side effects.^41^ Regarding the antifungal activity of alginate and its copolymer, *Candida albicans* was chosen as fungi species as it is the superior fountain of in function by fungi critically malady or immunocompromised patients.^42^ Moreover, the grafting of alginate by poly(*N*-vinyl imidazole) is exhibited antifungal activity against *Candida albicans* according to data from Table 2 and Fig. 7, which show that the inhibition zone diameter becomes 9 mm mg^−1^ in case of the grafted alginate instead of zero mm in case of sodium alginate. Poly(*N*-vinyl imidazole) is soluble in water and P*N*VI has significant antibacterial activity due to the existence of the imidazole ring.^43^ That effect could be explained by the good binding properties of the imidazole ring owing to the presence of nitrogen atoms of the imidazole ring (position 3) and this nitrogen atom is e-donor. Because of the e-donor of the nitrogen atom. The explained reason makes *N*VI have great function in protein–enzyme separation.^44–46^ The grafted copolymer alginate-*g*-P*N*VI with graft percent equal to 65%, and its antimicrobial activity was studied against the same microbial. Furthermore, alginate-*g*-P*N*VI (%*G* = 65%) has same behavior of alginate-*g*-P*N*VI (%*G* = 120%) against bacteria species, which are Gram-negative bacteria (*Escherichia coli* and *Neisseria gonorrhoeae*) and Gram-positive bacteria (*Bacillus subtilis*) as shown in Table 2. There is no significant improvement of antifungal properties of alginate by grafting percent equal 65% using *Candida albicans*.

**Table 2:** Inhibition zones diameters observed in case of alginate and its grafted copolymers NaAlg-*g*-P*N*VI with % = 65% and 120%

Control	DMSO	Inhibition zone diameter (mm mg^−1^ sample)			
		Bacterial species			Fungi
		(G^+^)	(G^−^)		
		*Bacillus subtilis*	*Escherichia coli*	*Neisseria gonorrhoeae*	*Candida albicans*
		0.0	0.0	0.0	0.0
Standard	Ampicillin: antibacterial	22	31	27	19
	Amphotericin B: antifungal				
Polymer	Sodium alginate	0.0	9	0.0	0.0
	NaAlg-*g*-P*N*VI (*G*% = 90%)	11	11	10	0.0
	NaAlg-*g*-P*N*VI (*G*% = 120%)	10	11	10	9

## 4. Conclusion

Sodium alginate was chemically modified through the grafting technique. The thermal behavior of grafted alginate showed a lower rate of degradation than native alginate. The prepared grafted copolymer was characterized with different techniques like (FT-IR) and thermal analysis. Scanning electron microscope (SEM) presented changes in the surface of sodium alginate by the grafting of *N*-vinyl imidazole to confirm the change in the morphology after the grafting process. The original sodium alginate and grafted copolymer NaAlg-*g*-P*N*VI have investigated their antimicrobial activity. The antimicrobial tests revealed that sodium alginate exhibited antimicrobial activity by *N*-vinyl imidazole grafting on its backbone.

## Author contributions

S. M. A. S. helps in preparing, investigation, methodology and characterization of the obtained materials. S. M. A. S. and M. F. S. contributed in discussed the results. Furthermore, S. M. A. S. and A. E. S. designed the research, contributed to supervising the work, discussed the results and wrote the manuscript. All the authors participated in writing, editing and revising the manuscript.

## Conflicts of interest

The authors declare no conflict of interest.

## Supplementary Material

RA-011-D1RA01874D-s001
