# Supplementary material for: Synthesis, characterization and antimicrobial activity applications of grafted copolymer alginate-g-poly(N-vinyl imidazole)
Source: RSC Adv. 2021 Mar 19;11(19):11541–8. doi: 10.1039/d1ra01874d (PMC8695916; doi:10.1039/d1ra01874d)
Supplement: RA-011-D1RA01874D-s001 [file RA-011-D1RA01874D-s001.pdf]

Electronic Supplementary Material (ESI) for RSC Advances.
This journal is © The Royal Society of Chemistry 2021

Supporting Information

Synthesis, characterization and antimicrobial activity applications of grafted copolymer alginate-g-poly (*N*-vinyl imidazole)

Soliman Mehawed Abdellatif Soliman[1,*] *Mohamed Fathi Sanad*[3] *and Ahmed Esmail Shalan*[2,4*]

[1] Chemistry Department, Faculty of Science, Cairo University, 12613 Giza, Egypt.
[2] Central Metallurgical Research and Development Institute (CMRDI), P.O. Box 87, Helwan, Cairo 11421, Egypt.
[3] FabLab, Centre for Emerging Learning Technologies (CELT), and Electrical Engineering department, the British University in Egypt (BUE), Cairo 11387, Egypt.
[4] BCMaterials, Basque Center for Materials, Applications and Nanostructures, Martina Casiano, UPV/EHU Science Park, Barrio Sarriena s/n, Leioa 48940, Spain.

[*]Corresponding author: (A. E. S.) E-mail: *ahmed.shalan@bcmaterials.net;*
a.shalan133@gmail.com
(S. M. A. S.) E-mail: *sabdellatif@sci.cu.edu.eg*

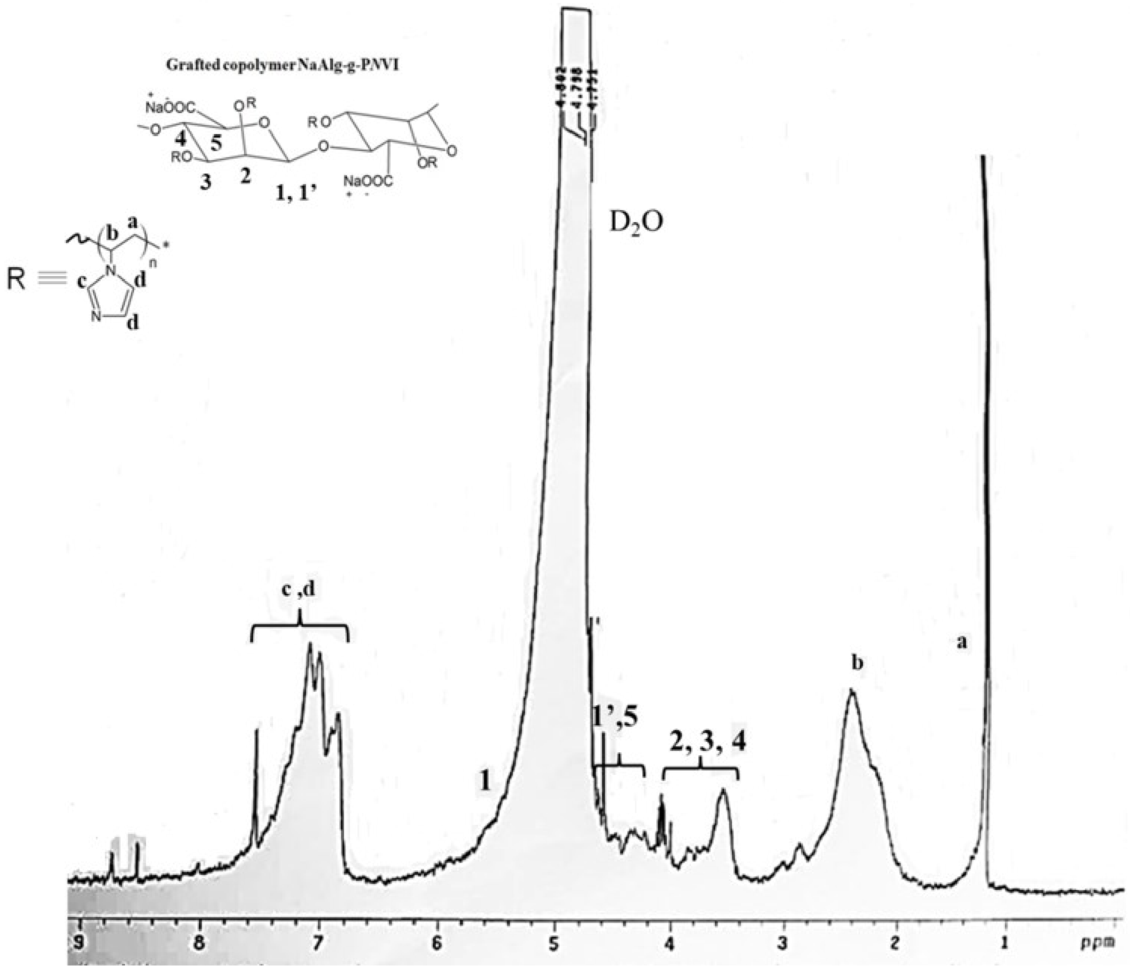

Figure S1. ^{1}H NMR spectrum of grafted copolymer Alginate-g-P*N*VI in D_2O.

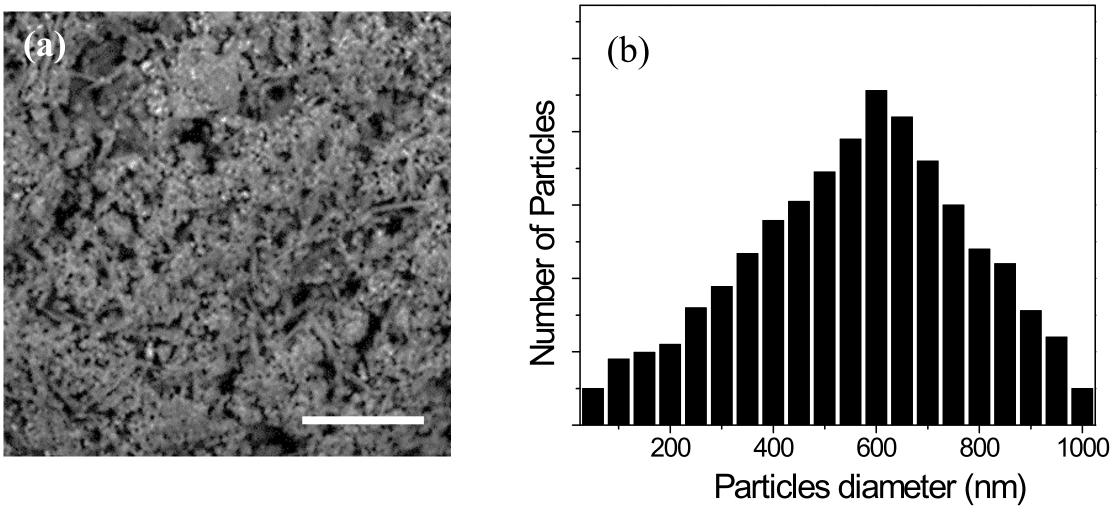

Figure S2. (a) SEM pictures and (b) particle size distribution of grafted copolymer NaAlg-g-P*N*VI
